# Investigation of the Absorption of Nanosized lamotrigine Containing Nasal Powder via the Nasal Cavity

**DOI:** 10.3390/molecules25051065

**Published:** 2020-02-27

**Authors:** Rita Ambrus, Péter Gieszinger, Róbert Gáspár, Anita Sztojkov-Ivanov, Eszter Ducza, Árpád Márki, Tamás Janáky, Ferenc Tömösi, Gábor Kecskeméti, Piroska Szabó-Révész, Csilla Bartos

**Affiliations:** 1 Institute of Pharmaceutical Technology and Regulatory Affairs, University of Szeged, Eötvös u. 6, H-6720 Szeged, Hungary; gizipet@gmail.com (P.G.); revesz@pharm.u-szeged.hu (P.S.-R.); bartoscsilla@pharm.u-szeged.hu (C.B.); 2 Department of Pharmacology and Pharmacotherapy, University of Szeged, Dóm tér 12, H-6720 Szeged, Hungary; gaspar@med.u-szeged.hu or; 3 Department of Pharmacodynamics and Biopharmacy, University of Szeged, Eötvös u. 6, H-6720 Szeged, Hungary; Ivanov.Anita@pharm.u-szeged.hu (A.S.-I.); ducza@pharm.u-szeged.hu (E.D.); 4 Department of Medical Physics and Informatics, University of Szeged, Korányi fasor 9, H-6720 Szeged, Hungary; marki.arpad@med.u-szeged.hu; 5 Department of Medical Chemistry, University of Szeged, Dóm tér 8, H-6720 Szeged, Hungary; janaky.tamas@med.u-szeged.hu (T.J.); tomosi.ferenc@med.u-szeged.hu (F.T.); kecskemeti.gabor@med.u-szeged.hu (G.K.)

**Keywords:** nasal powder, nose to brain delivery, lamotrigine, permeability, in vivo study

## Abstract

Nasal drug delivery has become a popular research field in the last years. This is not surprising since the nose possesses unique anatomical and physical properties. Via the nasal mucosa local, systemic, and directly central nerve systemic (CNS) effect is achievable. Powders have favorable physicochemical properties over liquid formulations. Lamotrigine (LAM) is an antiepileptic agent with a relatively mild side effect spectrum, but only available in tablet form on market. Reducing the particle size to the nano range can affect the bioavailability of pharmaceutical products. The aim of this article was to continue the work started, compare the in vitro properties of a nanonized lamotrigine containing nasal powder (nanoLAMpowder) and its physical mixture (PM) that were prepared by dry milling. Moreover, to study their trans-epithelial absorption to reach the blood and target the brain by axonal transport. Due to the dry milling technique, the particle size of LAM, their surface and also their structure changed that led to higher in vitro dissolution and permeability rate. The results of the in vivo tests showed that the axonal transport of the drug was assumable by both intranasal formulations because the drug was present in the brain within a really short time, but the LAM from the nanoLAMpowder liberated even faster.

## 1. Introduction

The nose has received considerable attention among the alternative drug administration routes in the last few decades [1,2]. Besides the most known local effect, systemic and central nervous system (CNS) effects can be achieved through the nasal mucosa. The systemic effect can occur if the active pharmaceutical ingredient (API) is absorbed into the systemic circulation via the nasal mucosa and at the right place, affects. This is the so-called “nose-to-blood” transport [3,4]. The CNS effect can be elicited in two ways. The first possibility is the “nose-to-brain” administration. In this case, the API accesses the CNS directly by axonal transport—through the trigeminal and olfactory nerve—as the CNS has free nerve endings in the olfactory bulb [5,6,7]. The other way is when the API gets into the systemic circulation and from there through the blood–brain-barrier (BBB) to the CNS [8,9,10]. Accordingly, the nose can be a great alternative administration route in the therapy of CNS diseases or when the target is in the CNS (e.g., brain tumors). Thus, in recent years, researchers have made some efforts to formulate drug delivery systems for the treatment of schizophrenia [11,12], epilepsy [13,14], Alzheimer’s disease [15,16,17,18], Parkinson’s disease [19,20], migraine [21], vaccination [22,23], and brain tumor [24]. In some cases the aim was to formulate nanosystems, that may be advantageous due to reduced particle size and increased particle surface, which can affect the absorption positively [25]. Our research group has also successfully formulated nanosized meloxicam and meloxicam potassium monohydrate containing nasal spray formulation for nasal drug delivery [26,27].

Nasal powder formulations have better physicochemical and microbiological stability over liquid formulations [28,29,30]. Thus, preservatives are not necessary, their shelf-life is longer, transportation is easier and packaging is more economical compared to liquid formulations [31]. By applying powder formulations, higher administration dose and better adhesion to the nasal mucosa can be achieved, that may cause better absorption through the barrier [32,33]. In our previous studies, nanosized lamotrigine (LAM) containing nasal powder was produced and developed by the Quality by Design method [34,35].

LAM is a second-generation, poor water-soluble antiepileptic drug from the phenyltriazine and the BSC II class with a relatively good side effect spectrum [36]. It is used for the treatment of epilepsy, Lennox-Gastaut syndrome and bipolar disease in monotherapy or as an adjuvant [37,38,39]. It is absorbed from the GI tract with no significant First Pass Effect (FPE). When taken orally, the API reaches its peak plasma concentration within 2.5 h. This can be slightly prolonged in the case of post-meal administration, but the extent of absorption remains unchanged. This quite late appearance at the point of effect reduces the chance of intervention in an incidental adverse event. Moreover, there are diseases (e.g., malabsorption, acute diarrhea), where an alternative dosage form is desirable. Though there have been some efforts to formulate an alternative dosage form for LAM, such as orally disintegrating or chewable tablets, they would not be fast enough to ensure a safe solution for unexpected seizures. That is why a more rapid solution is needed, which can be served by a nasal formulation [40,41,42,43,44,45,46].

Previously, a nanosized LAM containing nasal powder was produced, optimized and its preparation method was well established. As a continuation of our previous studies [34,35], the aim of this work was to determine the physicochemical properties, the in vitro characteristics and the in vivo absorption profile of a possible novel, nasal powder dosage form of LAM and compare it to the physical mixture as a reference with the hypothesis that smaller particle size results in higher absorption.

## 2. Results and Discussion

### 2.1. Determination of Particle Size and Image Analysis (SEM)

**Figure 1:**
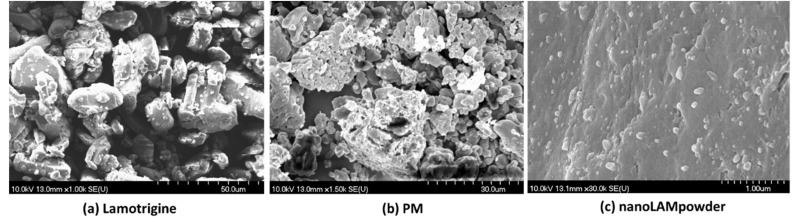
SEM pictures of the samples. In picture (**a**) raw LAM particles, in picture (**b**) the PM sample, and in picture (**c**) the nanoLAMpowder can be seen. Abbreviations: physical mixture (PM); nanonized lamotrigine containing nasal powder (nanoLAMpowder).

The particle size of LAM and the samples is shown in Table 1. The acceptable range that is required for nasal powders is between 5 and 45 µm [31]. The raw, crystalline LAM particles were in this acceptable, micrometric range, but they were aggregated, which could make the application of the API uncertain. This was proven by the SEM pictures (Figure 1). In Figure 1 picture (a) the above-mentioned micrometric LAM particles can be seen. In the case of the PM sample, the particle size of the product was smaller compared to nanonized lamotrigine containing nasal powder (nanoLAMpowder). The explanation of this phenomenon is that the PVA (polyvinyl alcohol) was milled itself in the physical mixture (PM), so the LAM did not influence the milling process. If LAM particles were there, they would have been able to prevent the particle size-reducing effect of the balls and the PVA particles on each other, which would result in a larger product particle size.

In the SEM picture of PM (Figure 1 picture (b)) the aggregation of the API particles can be observed, while in the nanoLAMpowder ((c) picture) sample they were nanosized particles with approximately 100 nm particle size. In Figure 1 picture (c) the LAM showed homogenous distribution on the surface of the PVA matrix with no sign of aggregation.

### 2.2. Structural Investigations

#### 2.2.1. Differential Scanning Calorimetry (DSC)

**Figure 2:**
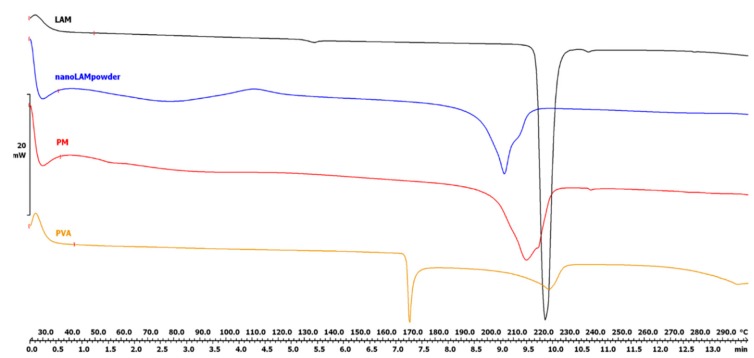
Differential scanning calorimetry (DSC) curves of the samples.

The DSC curves (Figure 2) show that the characteristic peak of LAM became less intensive due to the milling effect and the presence of PVA. This decrease in the area under the curve means that the crystalline habit of the drug decreased and the API became partly amorphous. This changed crystallinity degree—with the parallelly decreased particle size, increased surface and the presence of wetting agent, PVA— caused improved solubility value (0.0996 ± 0.0012 mg/mL vs. 0.1515 ± 0.0152 mg/mL) in water at 25 °C after 24 h as we could see in our previous study [32]. The obtained results could suggest a better dissolution and permeability rates since amorphous APIs could get into the aqueous phase easier, because of the lack of energy grid.

PVA (T_g_ = 34 °C) has two endothermic peaks at 169.51 °C and 222.74 °C. Since PVA is a mixture of crystalline and amorphous fractions, it is assumed that the first endothermic peak shows a structural change in the crystalline fraction and the second one presents the melting point of PVA [47].

#### 2.2.2. XRPD

**Figure 3:**
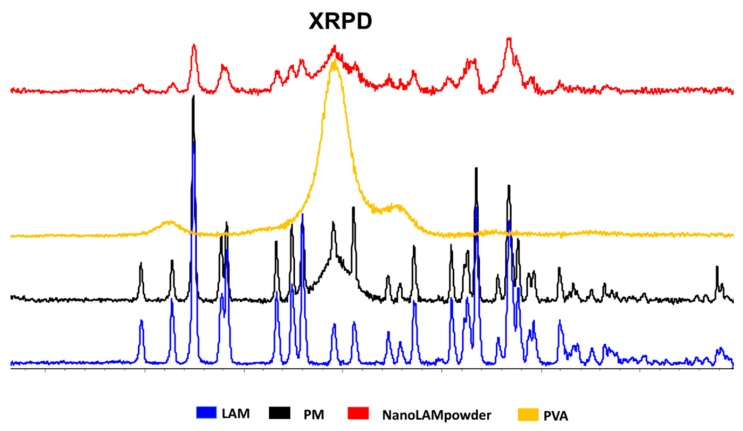
XRPD diffractograms of the samples.

Another way to investigate the degree of crystallinity is XRPD. Similarly to the results of the DSC measurement, the area under the curve was the smallest in the case of nanoLAMpowder (Figure 3). The crystallinity index was found to be 55.20% in the nanoLAMpowder sample, while it was 77.33% in the case of PM. The raw LAM was regarded as 100%. The results of the measurement confirmed the DSC results, which means that the LAM became partly amorphous due to the milling effect and the presence of PVA. The data obtained by both structural investigations are in agreement and confirm that this changed crystallinity structure may lead to higher in vitro dissolution and permeability rates [34], which can indicate better in vivo performance.

### 2.3. In Vitro Dissolution Study

**Figure 4:**
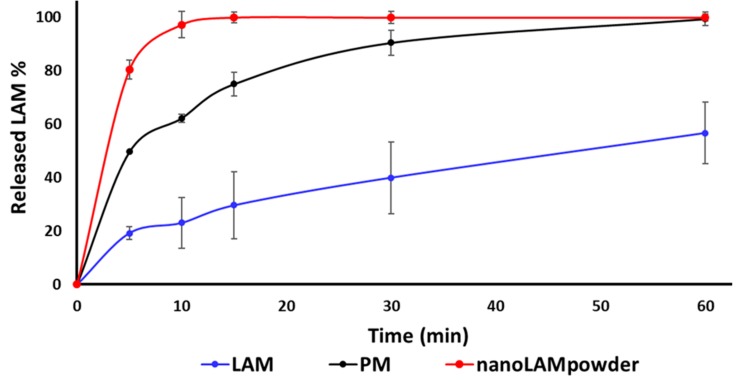
*In vitro* dissolution study of the samples in pH 5.60 buffer at 30 °C.

The results of the dissolution study confirmed our predictions (Figure 4.). The released amount of LAM was the lowest in the case of raw API during the whole investigation, while the PM performed remarkably good with 49.68% of LAM released after 5 min and 62.33% after 10 min, respectively. However, due to the small particle size, the high surface and the partly amorphous structure, 100% of LAM dissolved after 15 min from the nanoLAMpowder. Moreover, due to the presence of PVA, the efficiency of milling improved, it had a protective effect against aggregation, it could have a slight crystallization inhibitor effect and because of its water-soluble property, the unique, nanosized LAM particles could leave the polymer matrix rapidly. As the mucociliary clearance renews the mucosa in every 15 min, which can be extended for 20–25 min with different methods or additives, the results of nanoLAMpowder were promising for further studies.

### 2.4. In Vitro Permeability Study

**Figure 5:**
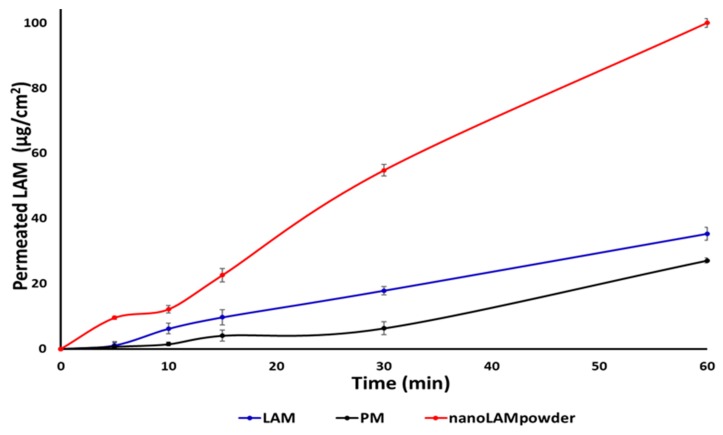
In vitro permeability study of the samples.

The horizontal diffusion test was carried out to investigate the in vitro permeability of the samples (Figure 5). It can be seen that the concentration of LAM was much higher in the acceptor phase in the nanoLAMpowder than in the other samples. This difference became extremely high at the end of the test. In contrast to the previous study, not large, but the raw LAM permeated from the donor phase to the acceptor in higher amounts than in the case of PM. The explanation of the results may be that the nanoparticles could leave the polymer matrix more effectively than when they were not nanonized.

These results are confirmed by the calculated Flux (J) and Permeability (K_p_) coefficient values in Table 2. The sample containing nanosized LAM had the best permeability, whereupon 100% of active substance passed to the acceptor phase after the end of the investigation. The reason for this high permeability can be explained by the function of PVA, better wettability [32], small particle size, and large particle surface. PVA can preserve the uniqueness of the particles which can liberate from its surface easily.

### 2.5. In Vivo Studies

**Figure 6:**
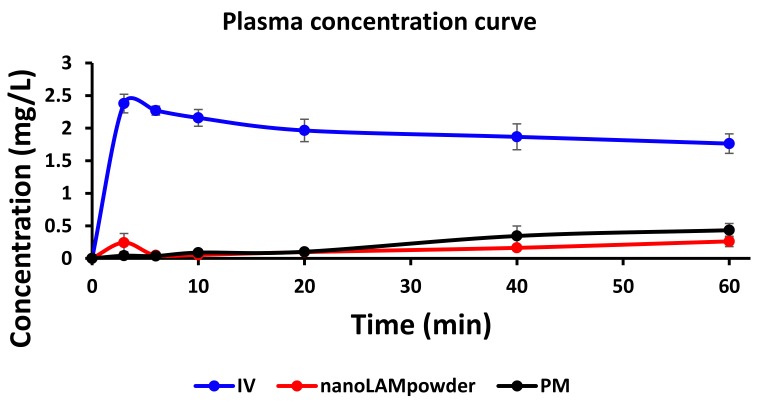
The concentration values of lamotrigine (LAM) in the blood plasma in the case of IV and intranasal administration.

The nanoLAMpowder formulation was compared to the PM and IV injection. In the case of IV and intranasal administration, the concentration values of LAM in the blood plasma vs. time profiles are shown in Figure 6. Compared to the nasal formulations, the plasma concentration of LAM was significantly higher in the IV group (2.38 ± 0.14 mg/L) in the first 3 min which provided the highest measured plasma concentration after the initiation of the injection. There were significant differences between the plasma concentrations in the case of nasal powder forms only in the first 3 min after application, however, these values were negligible compared to the IV administration. This means that there was no considerable difference in the absorption of the API into the systemic circulation by intranasal formulations.

**Figure 7:**
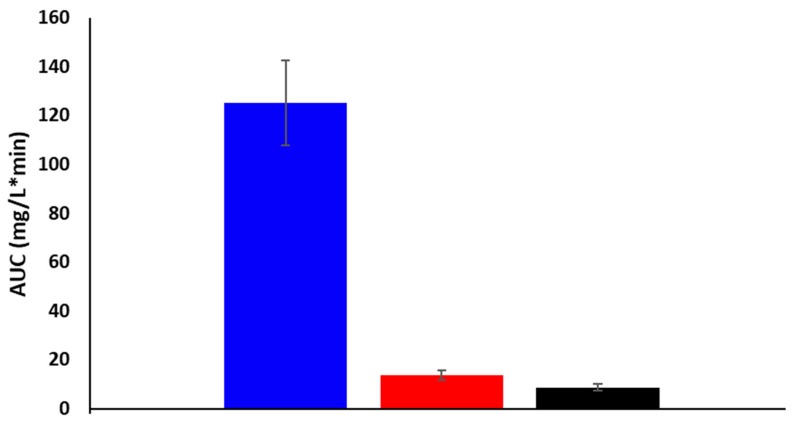
Plasma AUC values of powders and IV formulation.

The area under the curve (AUC) corresponds to the amount of drug absorbed into the systemic circulation during the investigated period. There were no remarkable differences between the plasma AUC values of powders (PM: 8.59 ± 1.35 mg·min/L; nano LAM powder: 13.63 ± 1.95 mg·min/L), however, they were significantly lower than the AUC value of IV administration (118.35 mg/L∙min) (Figure 7.). This could be explained with the 100% bioavailability of the drug after IV application, which can be achieved only in the case of intravascular administration.

**Figure 8:**
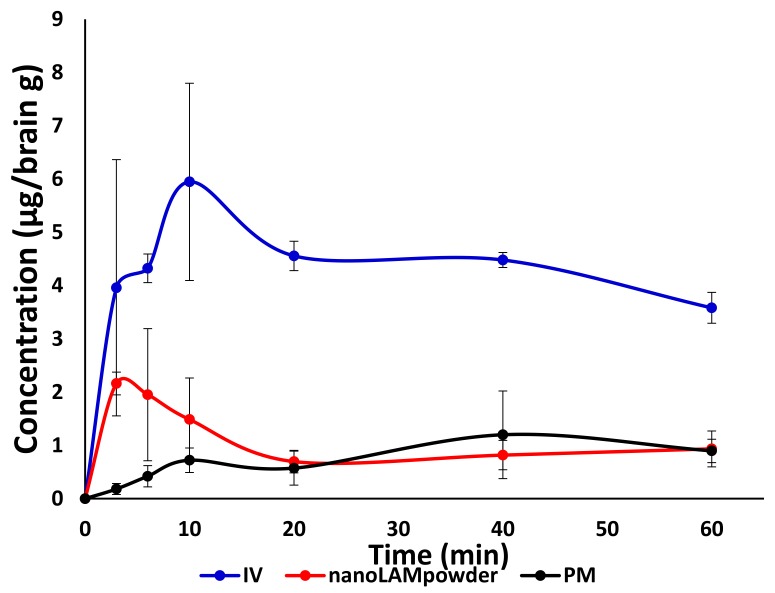
The concentration values of LAM in the brain samples.

The concentration values of LAM in the brain samples are shown in Figure 8. The application of nanoLAMpowder sample resulted in a significantly higher drug concentration (2.16 ± 0.21 µg/g) in brain tissues compared to the PM (0.18 ± 0.76 µg/ g) and IV injection (3.96 ± 2.41 µg/ g). The transport of the drug could be assumed by both intranasal formulations, because the drug was presented in the brain 3 min after administration, which period was not enough for the API to pass through the BBB after absorption into the systemic circulation from the nasal mucosa. In the case of PM, the drug-level in the brain was increased 40 min after application, which could be explained by the slower dissolution of microsized LAM particles and with the drug absorption through the BBB from the blood plasma.

**Figure 9:**
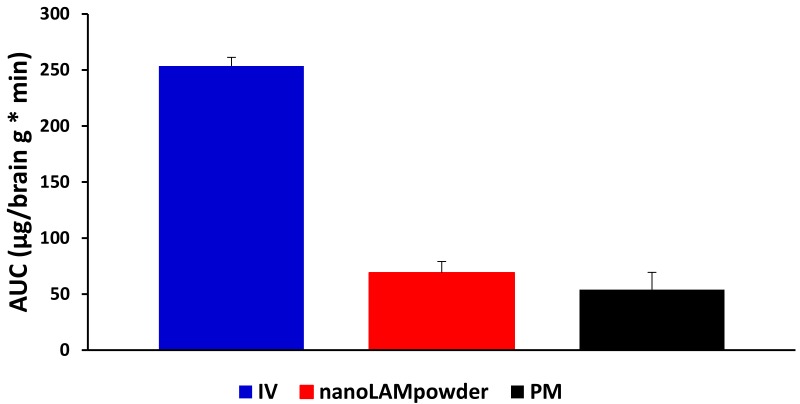
The cerebral AUC values of the formulations.

In terms of cerebral AUC values of the formulations (Figure 9), the administration of IV injection resulted in a higher AUC value (253.60 ± 7.66 μg·min/g) compared to the nasal formulations. This phenomenon could be elucidated with the 100% presence of the drug in the blood plasma after IV application, which may be absorbed through the BBB to target the brain. Due to the quick dissolution of nanoparticles, a higher amount of LAM could reach the brain directly by axonal transport in the case of nanoLAMpowder (69.05 ± 10.08 μg·min/g), resulting in almost two times higher AUC values than with the usage of PM (54.01 ± 15.39 μg·min/g).

To determine the utilization of the drug in the brain tissue, the absolute bioavailability was calculated, where the brain AUC—resulted by IV injection—was considered as 100% (Table 3). In the case of nanoLAMpowder, the absolute bioavailability of LAM was 39.84%, while in the case of PM it was only approximately 21%.

The cerebral drug targeting efficiency index (DTE) reflects the relative accumulation of the drug in the brain following intranasal administration as compared to systemic administration. DTE data were above 1.0 in case of both nasal powders, as the LAM could reach the brain tissues more efficiently via axonal transport, than through the systemic circulation. This resulted in remarkable absorption through the nasal mucosa directly into the CNS and parallelly resulted in poor transepithelial absorption into the systemic circulation.

## 3. Materials and Methods 

### 3.1. Materials

Lamotrigine, poorly water-soluble (0.17 mg/mL at 25 °C) was purchased from Teva Ltd. (Budapest, Hungary). Poly-vinyl alcohol (Mw = 27,000), water-soluble synthetic polymer—that was applied to stabilize the unique drug particles, thus improving their absorption—was supplied by ISP Customer Service GmBH (Cologne, Germany).

### 3.2. Sample Preparation

PVA was used as an additive during the sample preparation process to maintain the stability and individuality of LAM particles. NanoLAMpowder was produced as follows: 0.8 g PVA and 1 g LAM were mixed in a Turbula mixer (Turbula System Schatz; Willy A. Bachofen AG Maschinenfabrik, Basel, Switzerland) using 60 rpm for 10 min. After mixing, the sample was placed into a planetary ball mill (Retsch PM 100; Retsch, Neuhausen, Germany) and milled in a 50 mL capacity milling chamber for 1.5 h on 400 rpm with 10 steel balls (diameter 10 mm, the weight of each ball 4.02 g). In the case of the physical mixture (PM), PVA was milled for 1.5 h on 400 rpm and then—according to our previous experiments—it was mixed with non-milled LAM using the same Turbula mixer for 10 min on 60 rpm.

### 3.3. Determination of Particle Size and Image Analysis (SEM)

The particle size of the microparticles was characterized by using Leica Image Processing and Analysis System device (Leica Q500MC; Leica Microsystems, Wetzlar, Germany). The test parameters of 300 particles were their length, width, area, and district/convex perimeter.

The morphology and the size the LAM nanoparticles – that were on the surface of the polymer microparticles—were investigated by SEM (Hitachi S4700; Hitachi Ltd., Tokyo, Japan) at 10 kV. The samples were gold-palladium-coated (90 s) with a sputter coater (Bio-Rad SC502; VG Microtech, Uckfield, UK) using an electric potential of 2.0 kV at 10mA for 10 min. The air pressure was 1.3–13.0 mPa. Distributions of LAM particle diameter were obtained by analyzing SEM images with the ImageJ software (1.50i; Java 1.6.0_20 [32-bit]; Windows NT) environment using approximately 500 particles.

### 3.4. Structural Investigations

#### 3.4.1. Differential scanning calorimetry (DSC)

The thermal response of each product was measured using a differential scanning calorimeter (Mettler Toledo TG 821e DSC; Mettler Inc., Schwerzenbach, Switzerland). About 3–5 mg of powder was precisely weighed into DSC sample pans, which were hermetically sealed, and the lid was pierced. Each sample was measured in the temperature interval of 25–230 °C at a heating rate of 5 °C/min and at a rate of 5 °C/min under constant argon flow of 150 mL/min. Data analysis was performed using the STARe software (Version 9.30, Mettler Toledo; Mettler Inc., Schwerzenbach, Switzerland).

#### 3.4.2. X-Ray Powder Diffraction (XRPD)

The XRPD measurement was carried out with a BRUKER D8 advance X-ray powder diffractometer (Bruker AXS GmbH, Karlsruhe, Germany) with Cu⋅K λI radiation (λ = 1.5406 Å) and a VÅNTEC-1 detector (Bruker AXS GmbH, Karlsruhe, Germany). The powder samples were loaded in contact with a plane quartz glass sample slide with an etched square and measured. Samples were scanned at 40 kV and 40 mA. The angular range was 3°–40° 2θ, at a step time of 0.1 s and a step size of 0.007°. All manipulations, including Kα2 stripping, background removal and smoothing of the area under the peaks of the diffractograms, were performed using the DIFFRACplus EVA software. The crystallinity index (Xc) values were calculated based on the following formula, where A marks the area under the whole curve:

$$X_{c}=\;\frac{A_{\;crystalline}}{A{\;}_{crystalline\;}+\;A_{amorphous}}\;\times \;100\;$$

### 3.5. In Vitro Dissolution Study

The modified paddle method (USP dissolution apparatus, type II; Pharma Test, Hainburg, Germany) was used to examine the dissolution rate of LAM-containing co-milled nasal powders and determine the drug release profile from the samples. The test was carried out under nasal conditions for temperature and pH. 100 mL phosphate-buffered saline solution (PBS of pH 5.60 at 30 °C) was used as a medium, in which 108 mg of the samples were tested. The paddle was rotated at 50 rpm, and the sampling points were at 5 min, 10 min, 15 min, 30 min, and 60 min. At the mentioned sampling points, 2 mL of aliquot was taken from the media, which was replaced immediately to ensure the permanent media volume. In the beginning, the sampling point was more frequent, because the beginning of the investigation is more important as the mucociliary clearance renews the mucus every 15 min. The following sampling points offered extra information about the dissolution behavior of LAM. After filtration, the drug content of the aliquots was determined using spectrophotometry (Unicam UV/VIS Spectrophotometer, Unicam Ltd., Cambridge, UK) at 307 nm. The tests were carried out in triplicates.

### 3.6. In Vitro Permeability Study

The Side-Bi-Side™ (Grown Glass, New York, NY, USA) diffusion test was carried out in nasal conditions. Cellulose ester membranes with 0.45 μm pore diameter were soaked in isopropyl myristate, and the donor phase was tempered to 30 °C at a pH of 5.6. At determined moments (5, 10, 15, 30, and 60 min), 2 mL samples were taken from the acceptor phase, which volume was immediately replaced to maintain the permanent volume, thus the acceptor phase was diluted after every occasion. The acceptor phase had a pH of 7.4, and the concentration of the diffused drug was measured after filtration, spectrophotometrically at 307 nm (Unicam UV/Vis Spectrophotometer, Unicam Ltd., Cambridge, UK). The optical path length was 1 cm. The tests were carried out in triplicates.

### 3.7. In Vivo Studies

#### 3.7.1. IN Administration, Blood Sample Collection, and Brain Removal

The nanoLAMpowder and also the PM contained 0.555 mg LAM. This dose was administered into the right nostril of 160–180 g male Sprague–Dawley rats (*n* = 4) with a small spatula. The administration was carried out under isoflurane short anesthesia. As a control, IV injections of LAM solutions (IV LAM) containing 0.555 mg of API were given to rats (*n* = 4). At predetermined time points (3, 6, 10, 20, 40, and 60) after LAM administration, blood samples were collected by cardiac puncture into heparinized tubes under deep isoflurane anesthesia. Then the animals were sacrificed by decapitation, and brain tissues were quickly removed, rinsed in ice-cold PBS, divided into left and right hemispheres, weighed, and stored at –80 °C until assayed. The experiments were performed according to the EU Directive 2010/63/EU for animal experiments and were approved by the Hungarian Ethical Committee for Animal Research (permission number: IV/1247/2017).

#### 3.7.2. Plasma Sample Preparation

Rat plasma samples were stored at −80 °C until use. On the day of extraction, the samples were thawed, vortexed and 100 µL of plasma samples were placed into glass vials. After adding 20 µL internal standard solution (4 µg/mL, lamotrigine-13C3, d3 in methanol-water, 50:50, *v*/*v*), 20 µL methanol-water mixture (50:50, *v*/*v*) and 100 µL 2 M sodium hydroxide, the samples were vortexed. For the liquid-liquid extraction 1 mL, ethyl acetate was added to each glass and then was vortexed 3 times for 1 min. After centrifugation, 100 µL of the supernatant was transferred to a new 1.5 mL glass vial, then dried at room temperature using a gentle stream of nitrogen with an MD 200-2 sample concentrator (Hangzhou Allsheng Instruments CO., Ltd.; Hangzhou, China). The samples were resuspended in 100 µL of acetonitrile-containing formic acid (0.1 % *v*/*v*) and diluted with 0.1% formic acid to a final volume of 800 µL. 5 µL was injected into the LC-MS/MS system (Agilent; Santa Clara, CA, USA) for analysis.

Prior to extraction of the calibration points, 20 µL of a standard solution (0.1–32 µg/ mL) was added to 100 µL of pooled rat plasma instead of a methanol-water mixture. Quality control samples (low: 2.0 µg/mL, high: 16.0 µg/mL) were prepared similarly. The rest of the sample preparation process was the same as described above.

#### 3.7.3. Brain Tissue Sample Preparation

The rat brain was placed into 15 mL centrifuge tubes and 4 mL of water was added to 1 gram of brain tissue. The samples were homogenized on ice 2 times for 30 s with an ULTRA-TURRAX blade-type homogenizer (IKA^®^ Works, Inc; Wilmington, NC, USA) and for 30 s with a BioLogics Model 150VT ultrasonic homogenizer (BioLogics Inc, Manassas, VAUSA). The samples were stored at −80 °C until use. On the day of extraction, the samples were thawed, vortexed and 200 µL of these homogenates was placed into a 1.5 mL Eppendorf tube. After adding 20 µL internal standard solution (5 µg/mL, lamotrigine-13C3, d3 in methanol-water, 50:50, *v*/*v*), 20 µL methanol-water mixture (50:50, *v*/*v*) and 20 µL 20% (*w*/*v*) trichloroacetic acid (TCA), the samples were vortexed. After centrifugation at 10,000× *g* for 10 min at 20 °C, 100 µL of the supernatant was transferred into a 1.5 mL glass vial. 100 µL 4M sodium hydroxide was added to the samples. For the liquid-liquid extraction, 1 mL ethyl acetate was added to each glass vials and vortexed 3 times for 1 min. After centrifugation, 50 µL of the supernatant was transferred to a 96 well polypropylene plate and dried in a vacuum centrifuge at room temperature. The samples were resuspended in 25 µL of acetonitrile-containing formic acid (0.1 % *v*/*v*) and diluted with 0.1 % formic acid to a final volume of 185 µL, and 5 µL was injected into the LC-MS/MS system for analysis.

Prior to the extraction of the points of the calibration curve, 20 µL of standard solution (0.125–25 µg/ mL) was added to the pooled rat brain homogenate instead of a methanol-water mixture. Quality control samples (low: 1.0 µg/mL, high: 10.0 µg/mL) were prepared similarly. The rest of the sample preparation was the same as described above.

#### 3.7.4. LC-MS/MS

The liquid chromatography separation was performed using an Agilent 1100 Series high-performance liquid chromatograph (Agilent; Santa Clara, CA, USA). The separation was achieved using a Kinetex C18 (2.6 µm 100A, 50 × 2.1 mm) LC column (Phenomenex; Torrance, CA, USA). A C18 guard column was used before the analytical column. Water (A) and acetonitrile (B) both containing formic acid (0.1 % *v*/*v*) were used as mobile phases. The gradient program was used to elute components: gradient started at 13% B, increased linearly to 90% B in 3 min, kept at 90% B for 2 min, dropped to 13% B in 0.1 min and kept at 13% B for 2.9 min. The flow rate was set to 300 µL/min for the separation and 500 µL/min to wash and equilibrate the column. The autosampler and the column were maintained at room temperature. An injection volume of 5 µL was used for each analysis.

Samples were analyzed with a Q Exactive Plus quadrupole-orbitrap hybrid mass spectrometer (Thermo Fisher Scientific; Waltham, USA) equipped with a heated electrospray ion source (HESI) operating in positive mode with the following conditions: capillary temperature 256 °C, S-Lens RF level 50, spray voltage 3.5 kV, sheath gas flow 48, sweep gas flow 2 and auxiliary gas flow 11. Automatic gain control (AGC) setting was defined as 2 × 10^5^ charges and the maximum injection time was set to 100 ms. Collision energy (CE) was optimized and set at 31 eV for lamotrigine and lamotrigine-13C3, d3 (ISTD). The precursor to product ion transition of *m*/*z* 256.01→108.98 (qualifier), 256.01→210.98 (quantifier) for lamotrigine and *m*/*z* 262.04→110.99 (qualifier), 262.04→217.01 (quantifier) for ISTD were used for parallel reaction monitoring (PRM).

Data acquisition and processing were performed using Xcalibur™ Software (Thermo Fisher Scientific; Waltham, MA, USA).

#### 3.7.5. Sample Preparation of Rat Plasma and Brain

Calculations of the area under the time-concentration curve (AUC) and statistical analysis

The area under the curve (AUC) of the time (min)–concentration (mg/L) curves of each animal and the statistical analysis were performed with Prism 5.0 software (GraphPad Software Inc., La Jolla, CA, USA). All reported data are means ± SD. Student’s unpaired t-test was used to determine statistical significance. Changes were considered statistically significant at *p* < 0.05. The ratio of AUC value, after intranasal application in the brain in comparison with the AUC of IV administration (absolute bioavailability for brain—% abs. BA for the brain) was determined according to the formula:

$$\%abs.\;BA\;for\;brain=\;\frac{AUCbrain\;IN}{AUCbrain\;IV}\;\times \;100$$

Drug targeting efficiency (DTE)—relative exposure of the brain to the drug following intranasal administration vs. systemic administration—was calculated according to the following formula:

$$DTE=\;\frac{\left(\frac{AUCbrain}{AUC\;blood}\right)IN}{\left(\frac{AUCbrain}{AUCblood}\right)IV}$$

The value of DTE can range from −∞ to ∞, and the values higher than 1.0 indicate more efficient drug delivery to the brain following intranasal administration as compared to the systemic administration [26].

Calculations of the area under the time-concentration curve (AUC) and statistical analysis

The calculation of area under the curve (AUC) of the time (min)—concentration (µg/L) curves of each group of animals were performed with PKSolver add-in of Microsoft Excel (MS Office 2010, Reston, VA, USA) using non-compartmental analysis of data after extravascular input (model #101) of LAM [48]. The AUC values were calculated using the linear trapezoidal method. Because of the incomplete elimination of LAM, the following parameters were not determined: λ, t_1/2_, AUC_0-inf_, AUMC_0-inf_, V_d_, and Cl. All reported data are means ± SD.

## 4. Conclusions

The aim of this work was to determine and compare the absorption rate of the previously optimized sample (nanoLAMpowder) and the physical mixture (PM) in vivo with the hypothesis that smaller particle size results in higher absorption. The micrometric investigations showed that the LAM was in the nano range in the case of nanoLAMpowder, while it was aggregated in the PM sample. This data was confirmed by the SEM pictures where the nanosized and homogenous distribution of API was observed on the PVA matrix in nanoLAMpowder. The structural investigations showed partial amorphization in the structure of LAM, while the results of in vitro dissolution and permeability tests showed a rapid and high dissolution rate from the nanoLAMpowder. The results were heavily influenced by the presence of the water-soluble PVA, which improved the milling efficiency and protected the nanoparticles from aggregation. The in vivo investigation showed that the plasma concentration of LAM was significantly higher in the IV group during the test compared to the nasally administered samples. Moreover, there was no considerable difference in the absorption of LAM into systemic circulation in the case of intranasal powders. However, the application of nano LAM containing sample resulted in a significantly higher drug concentration (90.37 μg/g·min) in the brain tissues compared to the PM (48.64 μg/g·min). The axonal transport of the drug was assumable by both intranasal formulations because the drug was presented in the brain in a very short time after administration. This period could not be enough for the passing through the BBB after absorption to the systemic circulation from the nasal mucosa. Thus the hypothesis was confirmed as the nanosized LAM reached the CNS in larger quantities. All in all, it can be said that the nanoLAMpowder showed a fast and high amount of drug release during the investigation in a short time, which makes it possible for being a major breakthrough in the therapy of epilepsy.

## Figures and Tables

**Table 1:** The particle size of the products

	Particle Size of Product (µm)	Particle Size of LAM in the Products (nm)
LAM	6.57 ± 5.59	-
PM	16.39 ± 7.17	Aggregated
nanoLAMpowder	29.91 ± 15.85	97 ± 60

**Table 2:** The calculated Flux (J) and the permeability coefficient (K_p_) values.

	J (µg/cm^2^/h)	K_p_ (cm/h)
LAM	35.29	0.011
PM	27.11	0.008
nanoLAMpowder	100	0.030

**Table 3:** Calculated parameters of intranasal powders applying IV administration as a benchmark. AUC: Area under the curve.

	abs. BA for brain (%)	AUC_brain_/AUC_blood_	DTE
IV injection	100	2.02	1
nano LAM powder	39.84	5.06	2.49
PM	21.30	6.29	3.11
